# Walking Training Increases microRNA-126 Expression and Muscle Capillarization in Patients with Peripheral Artery Disease

**DOI:** 10.3390/genes14010101

**Published:** 2022-12-29

**Authors:** Natan D. da Silva, Aluisio Andrade-Lima, Marcel R. Chehuen, Anthony S. Leicht, Patricia C. Brum, Edilamar M. Oliveira, Nelson Wolosker, Bruno R. A. Pelozin, Tiago Fernandes, Cláudia L. M. Forjaz

**Affiliations:** 1 Exercise Hemodynamic Laboratory, School of Physical Education and Sport, University of São Paulo, São Paulo 05508-900, Brazil; 2 Sport & Exercise Science, James Cook University, Townsville, QLD 4811, Australia; 3 Cellular Molecular Exercise Physiology Laboratory, School of Physical Education and Sport, University of São Paulo, São Paulo 05508-900, Brazil; 4 Laboratory of the Biochemistry and Molecular Biology of Exercise, School of Physical Education and Sport, University of São Paulo, São Paulo 05508-900, Brazil; 5 Albert Einstein Israelite Hospital, São Paulo 05652-900, Brazil

**Keywords:** intermittent claudication, physical exercise, capillarization, microRNA, VEGF

## Abstract

Patients with peripheral artery disease (PAD) have reduced muscle capillary density. Walking training (WT) is recommended for PAD patients. The goal of the study was to verify whether WT promotes angiogenesis in PAD-affected muscle and to investigate the possible role of miRNA-126 and the vascular endothelium growth factor (VEGF) angiogenic pathways on this adaptation. Thirty-two men with PAD were randomly allocated to two groups: WT (*n* = 16, 2 sessions/week) and control (CO, *n* = 16). Maximal treadmill tests and gastrocnemius biopsies were performed at baseline and after 12 weeks. Histological and molecular analyses were performed by blinded researchers. Maximal walking capacity increased by 65% with WT. WT increased the gastrocnemius capillary-fiber ratio (WT = 109 ± 13 vs. 164 ± 21 and CO = 100 ± 8 vs. 106 ± 6%, *p* < 0.001). Muscular expression of miRNA-126 and VEGF increased with WT (WT = 101 ± 13 vs. 130 ± 5 and CO = 100 ± 14 vs. 77 ± 20%, *p* < 0.001; WT = 103 ± 28 vs. 153 ± 59 and CO = 100 ± 36 vs. 84 ± 41%, *p* = 0.001, respectively), while expression of PI3KR2 decreased (WT = 97 ± 23 vs. 75 ± 21 and CO = 100 ± 29 vs. 105 ± 39%, *p* = 0.021). WT promoted angiogenesis in the muscle affected by PAD, and miRNA-126 may have a role in this adaptation by inhibiting PI3KR2, enabling the progression of the VEGF signaling pathway.

## 1. Introduction

Peripheral artery disease (PAD) is a common manifestation of atherosclerosis that mainly affects the lower limbs’ arteries [1]. It is estimated that more than 200 million people worldwide have PAD [2]. The arterial occlusion present in PAD limits blood flow to the affected muscles, producing ischemia during walking and inducing pain in the legs during the effort that releases with rest (called intermittent claudication—IC) [1]. Therefore, PAD patients present with reduced walking capacity compared to paired healthy individuals [3].

Many pathological processes may contribute to the impaired walking capacity in PAD. Recued muscle capillary density or rarefaction [4,5] may result in ischemia as well as lower limb muscle metabolic change characterized by reduction in type I fibers [5,6]. As greater walking capacity in patients with PAD is directly associated with a greater muscle capillary-fiber ratio [5,7] and a greater proportion of type I muscle fibers [5], therapies that improve limb vascularization and muscle fiber composition are key to enhancing walking capacity in this population.

Walking training (WT) is considered a primary treatment for improving walking ability in PAD [8]. It has been proposed that angiogenesis induced by WT precedes walking improvements in PAD [9]. In a recent study [10], we observed that WT improved the level of gastrocnemius inflammation and oxidative stress in patients with PAD. Additionally, WT enhanced eNOS expression, which may reduce endothelial dysfunction and stimulate muscle capillarization [11]. However, the molecular mechanisms underlying these processes are still not fully understood.

**Figure 1:**
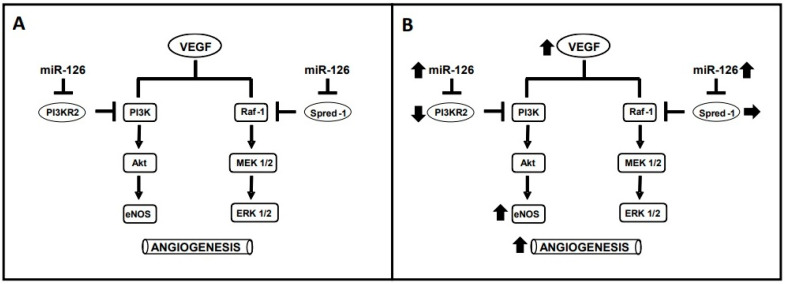
(**A**) Actions of miRNA-126 on the angiogenic pathways of the vascular endothelium growth factor (VEGF). (**B**) Effects of walking training on increasing (↑), decreasing (↓) or maintaining (→) each component of the angiogenic pathways of VEGF. PI3R2—phosphoinositol-2 kinase regulatory subunit 2; PI3K—Phosphoinoisitede 3-kinase; Akt—Protein kinase B; eNOS—nitric oxide synthase; SPRED-1—sprouty-related protein 1; RAF-1—threonine-protein kinase; MEK ½—mitogen-activated protein kinase kinase; ERK ½—extracellular signal-regulated kinases.

Vascular endothelium growth factor (VEGF) is important for promoting capillary growth in skeletal muscle [11]. Among the molecular mechanisms involved in VEGF angiogenic stimulation, microRNAs (miRNAs) are crucial contributors [12]. MiRNAs are small, non-coding RNAs that inhibit gene expression [13]. Amongst them, miRNA-126 is known for controlling angiogenesis by inhibiting two negative regulators of the VEGF signaling pathways, thereby promoting VEGF signaling (Figure 1A): (1) the sprouty-related protein 1 (SPRED-1), which inhibits the angiogenic pathway formed by RAF1-MEK-ERK; and (2) the phosphoinositol-2 kinase regulatory subunit 2 (PI3KR2), which inhibits the angiogenic pathway formed by PI3K-AKT-eNOS [14].

In a previous study, we showed that an acute bout of walking in patients with PAD increased the systemic expression of miRNA-126 and VEGF [15]. As chronic exercise adaptations may result from the constant physiological responses to the acute sessions [16], and an increase in systemic VEGF was reported after 12 weeks of WT in patients with PAD [17], it is possible to hypothesize that WT increases VEGF and miRNA-126 expression, thereby decreasing PI3KR2 and SPRED-1 inhibition of the VEGF-angiogenic pathways (i.e., RAF1-MEK-ERK 1/2 and PI3K-AKT-eNOS) to promote muscle capillarization. This study examined this hypothesis by verifying, in patients with PAD, whether WT promotes angiogenesis in the muscle affected by the disease and investigating a possible role for miRNA-126.

## 2. Materials and Methods

This study examined data from a randomized clinical trial approved by the Ethics Committee of Human Research at the University of São Paulo (process 667.382) and registered in the Brazilian Clinical Trials database (https://ensaiosclinicos.gov.br/, RBR-3pq58k, accessed on 4 June 2014). The outcomes of the clinical trial were published elsewhere [10], and the present study focused on the effects of WT on angiogenesis and its molecular regulation.

### 2.1. Study Design

Patients with PAD were divided into two groups (WT or control—CO) by a computer random number generator (htpps://www.randomizer.org, accessed on 2 August 2014). They were evaluated at baseline and after 12 weeks of intervention by blinded researchers. The main outcomes for the present study were: angiogenesis assessed by muscle capillary-fiber ratio; gene expression of miRNA-126; targets of miRNA-126 (PI3KR2 and SPRED-1), and VEGF.

### 2.2. Patients and Procedures

As the study explored extra data from a previously published randomized clinical trial, patient recruitment and detailed criteria for participation or exclusion can be assessed elsewhere [10]. Briefly, the study enrolled male patients with PAD at Rutherford stages 1–3 and presenting IC. They do not have any conditions that precluded walking, and were not receiving a β-blocker, non-dihydropyridine calcium channel antagonist, clopidogrel, or insulin.

The complete methodology of the study can also be assessed elsewhere [10]. Briefly, initially, the patients underwent preliminary exams to assure compliance to the study’s participation criteria. These procedures included a graded walking test until maximal claudication pain that was conducted on a treadmill and followed a specific protocol for PAD [18]. During the test, an ECG (Welch Allyn, Inc., Cardio Perfect MD, New York, NY, USA) was monitored, and the heart rate (HR) at the pain threshold (the beginning of claudication pain) was recorded [19]. Additionally, walking capacity was assessed by the distance walked until the onset of claudication (claudication onset distance, COD) and the maximal (total walking distance, TWD) leg pain experienced by the patients.

Then, for baseline assessment, on another day, muscle biopsies of the most affected lower limb were collected in a temperature-controlled laboratory (21–23 °C) following the procedures previously described [10]. Afterwards, the patients were randomized to their groups and received, twice per week for 12 weeks, their specific supervised interventions (WT—15 bouts of 2 min walking on a treadmill at the HR of the pain threshold, interpreted by 2 min of passive rest, and CO—stretching exercises) [10]. Finally, at least 48 h after the last training session, the maximal test and muscle biopsies were repeated.

### 2.3. Muscle Analyses

#### 2.3.1. Angiogenesis and Muscle Fiber Type Distribution

Frozen muscle samples were cut into 10 µm-thick sections using a cryostat (Criostat Micron HM505E, GMI, Ramsey, MN, USA). Immunohistochemistry was used to evaluate the fiber type distribution and capillary characteristics. Muscle sections were fixed with formalin 4% (no. HT501128; Sigma-Aldrich, St. Louis, MO, USA) for 10 min at room temperature, permeabilized in 0.2% Triton X-100 (no. 01-0407; Bio-Rad, Hercules, CA, USA) and 1% bovine serum albumin (BSA; no. E588; Amresco, Solon, OH, USA) diluted in phosphate buffered saline (PBS; no. P4417; Sigma-Aldrich) for 10 min, and blocked in 10% goat serum (G9023; Sigma-Aldrich) in PBS for 45 min. Glass slides were incubated with 1.5% goat serum in PBS and a solution containing the following primary antibodies for 1 h and 30 min at room temperature: (1) myosin heavy chain I (MHCI; 1:5000; no. ab11083; Abcam, Cambridge, UK) for muscle fiber type I; (2) laminin (1:100; no. ab7784; Abcam) for delimited muscle fibers; and (3) isolectin GS-IB4, Alexa Fluor 488 conjugate (1:100; no. I2411; Invitrogen, Waltham, MA, USA) for capillary staining combined with the wheat germ agglutinin (WGA); Alexa Fluor 555 conjugate (1:200; no. W32464; Invitrogen) for delimited muscle fibers. After washing with 0.2% Triton X-100 in PBS, the sections were incubated for 40 min in a dark room with PBS containing 1.5% goat serum and the respective fluorescent antibodies secondary to MHCI (1:500; Alexa Fluor 568 goat anti-mouse; no. A11004; Life Technologies, Carlsbad, CA, USA), laminin (1:500; Alexa Fluor 488 goat anti-rabbit; no. A11008; Life Technologies), and Hoechst (1:4000; nuclei visualization; no. H3569; Life Technologies). After washing, the slides were covered with coverslips using buffered glycerol (60% glycerol and 40% 0.1 M Tris·HCl pH 9.3). Fiber type distribution and capillary to fiber ratio were evaluated at 200× magnification using a computer attached to a microscope and connected to a photographic system (Leica Qwin; Leica Microsystems, Wetzlar, Germany). These images were further analyzed on a computerized digitizing unit (ImageJ software, version 1, National Institutes of Health, Bethesda, MD, USA). All analyses were conducted by a single observer who was blinded to the sample’s identity.

#### 2.3.2. RNA Extraction

Gastrocnemius muscle samples were homogenized in TRIzol, and RNA was isolated, according to the manufacturer’s instructions (Invitrogen Life Technologies, Grand Island, NY, USA). After extraction, the total RNA concentration was quantified using the NanoDrop Spectrophotometer (NanoDrop Technologies, Wilmington, DE, USA), and integrity was checked by EtBr-agarose gel electrophoresis.

#### 2.3.3. mRNA and miRNA Analysis by Real-Time Polymerase Chain Reaction

RNA was primed with 0.5 µg/µL oligo(dT) (Fermentas/Thermo Scientific Molecular Biology, Rockford, IL, USA) to generate first-strand DNA. Reverse transcription was performed using Revertaid M-MultV Reserve Transcriptase (Fermentas/Thermo Scientific Molecular Biology, Rockford, IL, USA). cDNA for miRNA analysis was synthesized from total RNA using gene-specific primers, according to the TaqMan miRNA assay protocol (Applied Biosystems, Carlsbad, CA, USA). Real-time quantifications of SPRED-1, PI3KR2, and VEGF mRNA were performed with a SYBRGreen PCR Master Mix using an ABI PRISM 7500 Sequence Detection System (Applied Biosystem, Carlsbad, CA, USA). Cyclophilin mRNA levels were used as a reference gene expression level. Primers were designed using the Primer3 software (http://primer3plus.com/cgi-bin/dev/primer3plus.cgi, accessed on 4 April 2020), and DNA sequence was obtained from GenBank. To accurately detect mature miRNA-126 (INV 2228), the real-time PCR quantification method was performed using the TaqMan miRNA assay protocol. MiRNA samples were normalized by evaluating RNU44 expression (Applied Biosystems, Carlsbad, CA, USA). Relative quantities of target gene expressions were compared after normalization to the reference gene value (ΔCT). Changes in mRNA and miRNA expression were calculated using the differences in ΔCT between the groups (ΔΔCT) and the equation 2^−ΔΔCT^. The results were expressed as a percentage of the baseline value obtained for the CO group.

### 2.4. Statistical Analysis

Since this study used a database from a randomized clinical trial [10], the sample size was calculated for the original trial and accepted for convenience in this investigation. Nevertheless, posteriori power analyses were performed and revealed a statistical power of >0.90 for the main outcome (muscle expression of microRNA-126). Normality of data distribution and homogeneity of variance were confirmed, respectively, by Shapiro-Wilk and Levene tests. Baseline groups’ characteristics were compared by *t*-tests or chi-squared tests. Walking capacity changes observed in each group were compared by *t*-tests. For the main outcomes, mixed two-way ANOVAs (Statsoft, Statistic for Windows 4.3, New York, NY, USA) were employed, with the stabling group (WT and CO) as the between main factor and phase (baseline and 12 weeks) as the within main factor. Newman-Keuls post-hoc tests were used when necessary. *p* ≤ 0.05 was considered significant. Data are presented as means ± SD in text and tables, while means ± SE were used in figures to a clearer view.

## 3. Results

The complete patients’ flowchart and group characteristics can be assessed elsewhere [10]. Briefly, 16 patients completed the study in each group, and their baseline characteristics regarding age, body mass index, ankle brachial index, and walking capacity were similar. Additionally, COD and TWD increased significantly only in the WT group (WT = +160 ± 163 vs. CO = −40 ± 74 m, *p* = 0.004, and WT = +375 ± 223 vs. CO = +39 ± 59 m, *p* < 0.001, respectively).

**Figure 2:**
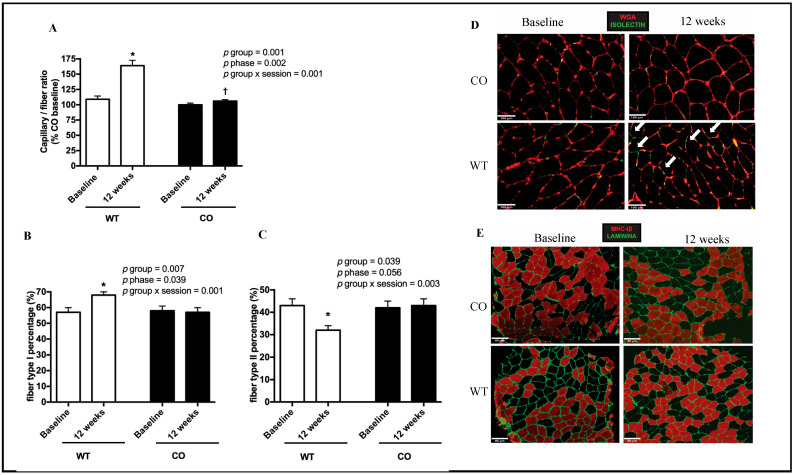
Muscle capillary-fiber ratio and percentage of muscle fiber types I and II assessed at baseline and after 12 weeks in the control (CO; *n* = 8) and walking training (WT; *n* = 8) groups. (**A**–**C**) present mean ± SE values. (**D**,**E**) show, respectively, representative figures of muscle capillary-fiber ratios and muscle fiber type percentages. White arrows indicate an increase in capillaries, * denotes a difference from the baseline within the group (*p* < 0.05), † denotes a difference from WT at 12 weeks (*p* < 0.05).

After the interventions, the muscle capillary-fiber ratio (WT = 109 ± 13 vs. 164 ± 21 and CO = 100 ± 8 vs. 106 ± 6%, *p* < 0.001, Figure 2A) and muscle fiber type I percentage (WT = 57 ± 9 vs. 68 ± 6 and CO = 58 ± 8 vs. 57 ± 8%, *p* = 0.001, Figure 2B) increased only for the WT group. In contrast, muscle fiber type II percentage decreased significantly only for this group (WT = 43 ± 9 vs. 32 ± 6 and CO = 42 ± 8 vs. 43 ± 8%, *p* = 0.003; Figure 2C). Illustrative representations of muscle structure assessed in each group at baseline and after 12 weeks are shown in Figure 2D,E.

**Figure 3:**
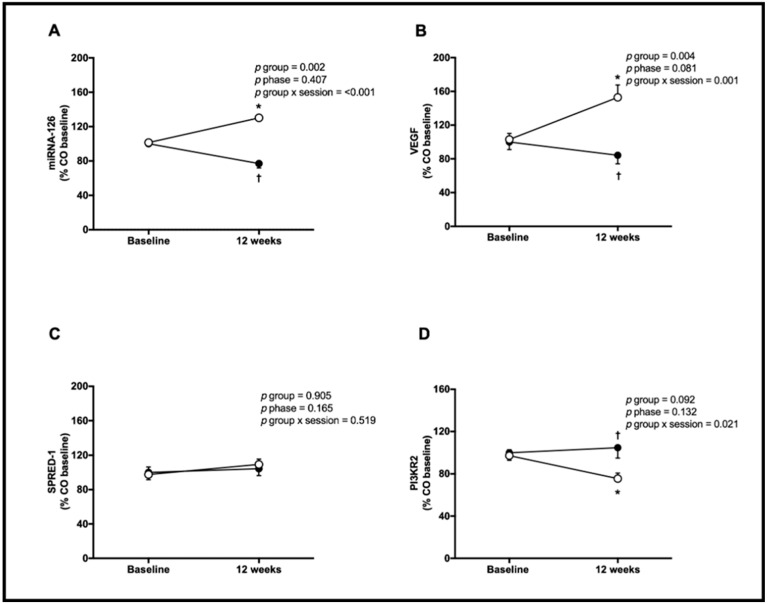
Muscle expression of microRNA-126 (miRNA-126, (**A**)), vascular endothelial growth factor (VEGF, (**B**)); sprouty-related protein 1 (SPRED-1, (**C**)) and phosphoinositol-3 kinase regulatory subunit 2 (PI3KR2, (**D)**) assessed at baseline and after 12 weeks for the control (CO; *n* = 16, black circles) and walking training (WT; *n* = 16, white circles) groups. Data: mean ± SE. * denotes a difference from baseline within the group (*p* < 0.05). † denotes a difference from WT at 12 weeks (*p* < 0.05).

Muscle expression of miRNA-126 increased for the WT group and decreased for the CO group following the interventions (WT = 101 ± 13 vs. 130 ± 5 and CO = 100 ± 14 vs. 77 ± 20%, *p* < 0.001; Figure 3A). VEGF increased for the WT group only (WT = 103 ± 28 vs. 153 ± 59 and CO = 100 ± 36 vs. 84 ± 41%, *p* = 0.001; Figure 3B), while muscle SPRED-1 expression showed no significant change statistically for both groups (WT = 98 ± 35 vs. 109 ± 25 and CO = 100 ± 34 vs. 104 ± 32%, *p* = 0.519, Figure 3C). In contrast, muscle PI3KR2 expression decreased (WT = 97 ± 23 vs. 75 ± 21 and CO = 100 ± 29 vs. 105 ± 39%, *p* = 0.021, Figure 3D) only for the WT group.

## 4. Discussion

The main findings of the study were that WT in patients with PAD improved walking capacity, promoted angiogenesis, and increased the muscular expression of VEGF and miRNA-126, enhancing the PI3K/Akt/eNOS angiogenic pathway.

Walking capacity is an important indicator of mobility and predicts mortality risk in patients with PAD [20]. In the present study, 12 weeks of WT increased the patient’s walking capacity by 65%, which is similar to previous studies [21] and may represent a reduction of 22% in mortality risk [22]. While an important health benefit for patients, the mechanisms of this WT-induced improvement have been unclear.

Previously, imaging studies demonstrated lower microvascular flow in the affected muscles of patients with PAD [23,24] that may contribute to their poor walking ability. Additionally, Robbins et al. [7] reported that lower local capillary density was directly associated with lower peak walking time in PAD. As expected, in the present study, WT increased the capillary-fiber ratio in the PAD-affected muscle, with this induced angiogenesis potentially contributing to the improvement of walking capacity. Additionally, the WT-induced angiogenesis may have resulted in greater skeletal muscle perfusion and modified muscle fiber composition [25], as the current patient’s lower limbs presented a greater proportion of type I fibers after the WT period. This adaptation may have significant clinical importance by minimizing the anaerobic metabolism dominance commonly observed in patients with PAD that limits their walking capacity [5,6].

Exercise is considered an efficient angiogenic stimulus by enhancing VEGF pathways. Along this line, an acute bout of exercise was reported to increase circulating and calf muscle VEGF mRNA in patients with PAD [15,26]. Additionally, regular WT was shown to increase resting levels of circulating VEFG in patients with PAD [17,27]. The present study extends upon this chronic training finding by directly assessing the muscle affected by the disease and observing increased local VEGF expression following WT. This expression potentially promoted the increase in capillarization, stimulating the localized change in muscle fiber composition and consequent increase in walking tolerance.

Besides the fact that WT induced greater VEGF expression, angiogenesis, and walking capacity in patients with PAD, the key novelty of the present study was the corresponding increase in miRNA-126 and PI3KR2, the target of miRNA-126, suggesting the participation of miRNA-126 in the VEGF signaling pathway of PI3K/Akt/eNOS. Along this line, data published before showed an increase in eNOS [10]. miRNA-126 involvement in angiogenesis has been proven by experimental studies [14,28] and may involve the inhibition of SPRED-1 and/or PI3KR2 [14,28]. In the present study, SPRED-1 expression was not changed by WT, while PI3KR2 expression was decreased, supporting the role of the PI3K-AKT-eNOS pathway in the angiogenic effect of WT. Therefore, regular WT may increase miRNA126 to inhibit PI3KR2 and enable the progression of the PI3K-AKT-eNOS signalizing pathway (Figure 1B).

This greater miRNA-126 expression after WT confirms prior results following an acute bout of walking in patients with PAD [15], after regular aerobic training in other human populations [29], and after training in rats’ skeletal and cardiac muscles [30,31]. Importantly and to the best of our knowledge, this is the first study to investigate the chronic effect of WT on the muscular expression of miRNA-126, VEGF, SPRED-1 and PI3KR2 in patients with PAD. The current novel outcomes support the involvement of miRNA-126 and the PI3K-AKT-eNOS pathway in the vascular adaptations induced by WT in PAD. 

### Study Limitations

While producing novel results for WT in PAD, this study had some limitations that should be acknowledged. It only included males, which limits extrapolation of the results to women. Future studies should confirm the current results in females to elaborate upon the mechanistic benefits of WT for all PAD patients. Additionally, only muscle capillarization, fiber type composition, and angiogenic markers were assessed with examination of muscle blood flow; future research may possibly extend the understanding of the mechanisms for WT-induced improvements in walking capacity in patients with PAD. Furthermore, only miRNA-126 was evaluated, with other miRNAs potentially playing a role in angiogenesis and WT-induced improvements. Examination of a range of miRNAs in future studies may confirm the complex nature of WT-induced angiogenesis in patients with PAD. Biopsies performed in these patients were used for morphological and genetic evaluations, allowing the analysis of the regulatory pathway mediated by microRNA-126-PI3KR2-VEGFa-eNOS that is involved in the angiogenic response to exercise training [30,32]. Nevertheless, protein analyses of other crucial markers of the angiogenic pathway need to be evaluated by future studies, since eNOS is largely regulated at the level of phosphorylation, and the protein analyses of other VEGF isoforms and their receptors may bring relevant results for better understanding the exercise effects on peripheral artery disease.

## 5. Conclusions

In conclusion, 12 weeks of WT promoted angiogenesis within the lower limb muscles affected by PAD. The WT-induced angiogenesis likely resulted from a greater expression of miRNA-126 that inhibits PI3KR2, enabling the progression of the VEGF angiogenic signaling pathway of PI3K-AKT-eNOS.

## Data Availability

The data underlying this article will be shared upon request from the corresponding author.
